# Factors Associated With Residual Hyperlipidemia Among Statin-Treated Adults With Diabetes and Atherosclerotic Cardiovascular Disease: A Cross-Sectional Analysis of National Health and Nutrition Examination Survey 2005 to 2018

**DOI:** 10.7759/cureus.107319

**Published:** 2026-04-19

**Authors:** Helen Oletu, Akinyele Oladimeji, Emeka K Okobi, Steve Okwu, Adewale A Kuye, Francis Okobi, Azeberoje Osueni, Onyekachi O Anya, Kwasi A Opoku, Emmanuella E Ogelebor

**Affiliations:** 1 Internal Medicine, North Knoxville Medical Center, Powell, USA; 2 Family Medicine, Alberta Health Services, Edmonton, CAN; 3 Maxillofacial Surgery, Ahmadu Bello University Teaching Hospital, Zaria, NGA; 4 Cardiothoracic Surgery, Beacon Hospital, Dublin, IRL; 5 Public Health, Imperial College London, Toronto, CAN; 6 Research and Development, Covance, Hanover, USA; 7 Gastroenterology and Hepatology, Lagos University Teaching Hospital, Lagos, NGA; 8 Internal Medicine, Legacy Salmon Creek Medical Center, Vancouver, USA; 9 Internal Medicine, St. Theresa's Hospital, Nkoranza, GHA; 10 Internal Medicine, Kwame Nkrumah University of Science and Technology, Kumasi, GHA

**Keywords:** atherosclerotic cardiovascular disease, diabetes, nhanes, non-high-density lipoprotein, residual hyperlipidemia, statins

## Abstract

Background: Residual hyperlipidemia remains a concern among statin-treated individuals with diabetes and atherosclerotic cardiovascular disease, despite established lipid-lowering strategies. Persistent elevation of atherogenic lipoproteins contributes to continued cardiovascular risk in this population.

Objective: This study aimed to identify the demographic and clinical factors associated with residual hyperlipidemia among statin-treated adults with diabetes and atherosclerotic cardiovascular disease in the United States.

Methods: This cross-sectional study used data from the National Health and Nutrition Examination Survey 2005 to 2018. The study included adults aged 18 years and older with diabetes, atherosclerotic cardiovascular disease, and statin use. Residual hyperlipidemia was defined using non-high-density lipoprotein cholesterol greater than or equal to 100 mg/dL as a proxy for low-density lipoprotein cholesterol targets. Survey-weighted descriptive analyses and multivariable logistic regression were performed to assess associations.

Results: A total of 464 participants were included, representing a weighted population of 2,179,930. Residual hyperlipidemia was common in this group. Higher body mass index was associated with increased odds of residual hyperlipidemia (aOR=1.06; 95% CI: 1.02-1.10; p=0.006), while increasing age was associated with lower odds (aOR=0.94; 95% CI: 0.91-0.97; p=0.001). Other demographic and clinical variables were not significantly associated.

Conclusion: Residual hyperlipidemia remains prevalent among high-risk individuals despite statin therapy. These findings support the need for broader lipid assessment and targeted strategies to address persistent lipid abnormalities.

## Introduction

Atherosclerotic cardiovascular disease (ASCVD) that encompasses coronary artery disease (CAD), cerebrovascular disease, and peripheral arterial disease (PAD) is the major cause of mortality and morbidity in diabetic patients with diabetes [1,2]. High levels of low-density lipoprotein cholesterol (LDL-C) rank among the key modifiable risk factors in the development of ASCVD [3]. Statins have a critical and non-substitutable role in ASCVD prevention and management [4]. Patients with diabetes exhibit a higher prevalence of lipid abnormalities and are therefore at increased risk of ASCVD. Statin therapy has been shown to reduce ASCVD events in both coronary and non-coronary vascular disease populations [5].

Statins are used in the treatment of both total cholesterol and LDL-C and, less effectively, triglycerides and raise the level of high-density lipoprotein cholesterol (HDL-C) by a small to moderate amount [6]. Despite statin therapy, a high number of individuals still experience residual dyslipidemia alongside non-HDL-C and or apolipoprotein (ApoB) concentrations that do not reach the recommended levels [7]. Statins reduce cholesterol levels and exert anti-inflammatory effects, leading to significant reductions in cardiovascular events and their widespread use in both primary and secondary prevention of ASCVD [8]. Statin therapy is effective in lowering the incidence of major coronary events, coronary revascularization, and stroke by approximately a fifth of the five-year incidence with every mmol/L reduction in LDL-C levels, mostly regardless of the starting lipid profile or other presenting features [9]. Nevertheless, a substantial risk of cardiovascular disease events still exists even when a person is on statins [10].

It also seems that there are significant sex-, race-, and age-dependent disparities specific to statin adherence used in the primary prevention of cardiovascular diseases [11]. The adherence among women and racial and ethnic minority groups is always lower than in White adults, especially among Black and Hispanic adults [12]. Black and Hispanic patients have less adherence to statins, which is associated with racial/ethnic differences in cardiovascular mortality [13].

The residual burden of hyperlipidemia could be especially strong among people with diabetes and ASCVD [14]. Complex lipid abnormality in diabetes is characterized by elevated triglycerides, low levels of HDL-C, and a qualitative alteration in LDL particles, which increases atherogenicity [15]. All these metabolic imbalances, combined with the possible difficulty in reaching treatment compliance and even intensification, make the persistent lipid abnormalities even with statin therapy [16].

Lipid control and statin use practices among different populations have been examined before, but many studies have concentrated on a specific clinical environment or only on a cohort and not necessarily on national trends [17]. Moreover, fewer of the studies have specifically addressed the determinants of residual hyperlipidemia in statin-treated individuals with not only diabetes but also established ASCVD, who face very high risks of recurrent cardiovascular deaths [18]. These factors have to be understood on the level of the entire population to inform clinical decision-making and the strategies to be taken for the health of the population [19].

This study utilizes the National Health and Nutrition Examination Survey (NHANES) to offer quality findings. The NHANES offers a powerful yet nationally representative data that incorporates both extensive clinical and laboratory details and demographic data [20]. The objective of this study is to identify the demographic and clinical factors associated with residual hyperlipidemia (LDL-C ≥70 mg/dL) among statin-treated adults with diabetes and ASCVD in the United States using NHANES data. Additionally, the study will add to the existing knowledge on residual hyperlipidemia among statin-treated adults with diabetes and ASCVD.

## Materials and methods

Study design and data source

This study was a cross-sectional analysis using data from the NHANES conducted from 2005 to 2018 [21]. NHANES is a nationally representative survey of the noninstitutionalized US population that uses a complex, multistage probability sampling design. Data were collected through standardized interviews, physical examinations, and laboratory assessments. 

To account for the complex sampling design of NHANES and generate nationally representative estimates, survey weights, strata, and primary sampling units were incorporated into all analyses. Because multiple NHANES cycles (2005-2018) were combined, the examination sample weights (WTMEC2YR) were adjusted by dividing by the number of included cycles (seven cycles), consistent with NHANES analytic guidelines. A 14-year combined weight variable (wt_14yr) was therefore created as WTMEC2YR/7. Survey design was specified using the masked variance units (SDMVPSU) as primary sampling units and SDMVSTRA as strata. All analyses were conducted using Taylor series linearization with centered single-unit strata to ensure valid variance estimation.

Study population

The analytic sample included adults aged 18 years and older with diabetes and ASCVD who were receiving statin therapy. Diabetes was defined using self-report and laboratory measures, and ASCVD was defined based on self-reported history of coronary heart disease, myocardial infarction, or stroke. Statin use was identified from prescription medication data based on the reported use of atorvastatin, simvastatin, rosuvastatin, pravastatin, lovastatin, fluvastatin, or pitavastatin, including combination formulations containing these agents. After applying these criteria, 868 participants met the study inclusion criteria. Following the exclusion of participants with missing data on variables included in the analysis, the final analytic sample consisted of 464 individuals, corresponding to a weighted population of 2,179,930.

Variables and measures

The primary outcome was residual hyperlipidemia, initially defined as LDL-C greater than or equal to 70 mg/dL, consistent with the recommended targets for high-risk populations. However, LDL-C in NHANES is available only in the fasting subsample, which substantially reduced the analytic sample size to 79 participants after applying the study inclusion criteria. To preserve the study objective while ensuring adequate sample size, non-HDL-C was used as a proxy measure of atherogenic lipoprotein burden. Residual hyperlipidemia was therefore operationalized as non-HDL-C greater than or equal to 100 mg/dL. Non-HDL-C was calculated as total cholesterol minus HDL-C. This threshold corresponds to the recommended LDL-C target of less than 70 mg/dL and is supported by lipid management guidelines that recognize non-HDL-C as a secondary target reflecting total atherogenic lipoproteins [22,23]. In addition, non-HDL-C does not require fasting measurements, allowing the inclusion of a larger and more representative sample. Demographic variables included age, sex, race and ethnicity, and education level. Socioeconomic status was assessed using the income-to-poverty ratio. Clinical variables included body mass index and chronic kidney disease. Body mass index was given as kg/m². Chronic kidney disease was defined as an estimated glomerular filtration rate less than 60 mL/min/1.73 m², calculated using the Chronic Kidney Disease Epidemiology Collaboration 2021 creatinine equation [24].

Missing data

After applying the study inclusion criteria, missing data were present for several variables. The income-to-poverty ratio had 7.83% missing values, education had 0.23% missing values, body mass index had 4.03% missing values, and the outcome variable had 0.12% missing values. A complete-case analysis approach was used, and participants with missing data on variables included in the analysis were excluded.

Statistical analysis

All analyses accounted for the complex survey design of NHANES, including sampling weights, strata, and primary sampling units. Survey weights were adjusted to reflect the combined survey cycles. Descriptive statistics were used to summarize participant characteristics. Continuous variables were presented as means with standard deviations, and categorical variables were presented as weighted counts and column percentages. Group comparisons by residual hyperlipidemia status were conducted using survey-weighted t-tests for continuous variables and survey-adjusted chi-squared tests (Rao-Scott corrected), reported as design-based F statistics, for categorical variables. Multivariable logistic regression was used to evaluate factors associated with residual hyperlipidemia. Adjusted odds ratios with 95% confidence intervals were reported. All analyses were performed using Stata Version 18 (StataCorp LLC, College Station, Texas, United States) [22].

Ethical considerations

NHANES data are publicly available and deidentified. The survey protocols were approved by the National Center for Health Statistics Research Ethics Review Board, and written informed consent was obtained from all participants. This secondary analysis of publicly available data did not require additional institutional review board approval.

## Results

Table 1 presents the baseline characteristics of the study population stratified by residual hyperlipidemia status.

**Table 1:** Baseline characteristics by residual hyperlipidemia status Values are survey-weighted counts and column percentages. Continuous variables are presented as mean±standard deviation and compared using survey-weighted t-tests. Categorical variables are compared using survey-adjusted chi-squared tests (Rao-Scott correction), reported as design-based F statistics. P-values account for the complex survey design. The asterisk (*) denotes statistical significance (p<0.05). CKD was defined as an eGFR <60 mL/min/1.73 m². CKD: chronic kidney disease; eGFR: estimated glomerular filtration rate; GED: general educational development; HDL: high-density lipoprotein The table was generated by the authors using Stata Version 18 (StataCorp LLC, College Station, Texas, United States) [22].

Characteristic	Non-HDL <100 mg/dL (no residual hyperlipidemia; N=745,339)	Non-HDL ≥100 mg/dL (residual hyperlipidemia; N=1,434,591)	Test statistic	P-value
Age (years), mean±SD	71.25±9.56	65.35±9.17	t=5.05	<0.001*
Income-to-poverty ratio, mean±SD	2.70±1.67	2.70±1.54	t=0.01	0.996
eGFR (mL/min/1.73 m²), mean±SD	69.01±24.93	75.55±22.95	t=-1.91	0.061
Body mass index (kg/m²), mean±SD	30.75±6.40	33.96±6.76	t=-4.90	<0.001*
Gender, n (%)				
Male	456,823 (61%)	902,407 (63%)	F=0.04	0.843
Female	288,516 (39%)	532,184 (37%)		
Race/ethnicity, n (%)				
Mexican American	28,722 (4%)	57,115 (4%)	F=1.10	0.356
Other Hispanic	30,607 (4%)	61,643 (4%)		
Non-Hispanic White	529,452 (71%)	1,061,810 (74%)		
Non-Hispanic Black	80,505 (11%)	115,985 (8%)		
Non-Hispanic Asian	44,771 (6%)	44,557 (3%)		
Other race	31,282 (4%)	93,481 (7%)		
Education, n (%)				
Less than high school	157,451 (21%)	317,645 (22%)	F=0.62	0.517
High school graduate/GED	216,139 (29%)	490,679 (34%)		
College or above	371,749 (50%)	626,267 (44%)		
CKD (eGFR <60), n (%)				
No CKD	442,842 (59%)	1,061,567 (74%)	F=6.00	0.017
CKD	302,497 (41%)	373,024 (26%)		

According to the findings from the table above, participants with residual hyperlipidemia had a lower mean age compared with those without residual hyperlipidemia, 65.35 (9.17) years versus 71.25 (9.56) years, with a statistically significant difference (p<0.001). Body mass index was higher among participants with residual hyperlipidemia, 33.96 (6.76) kg/m², compared with 30.75 (6.40) kg/m², and this difference was statistically significant (p<0.001). The income-to-poverty ratio did not differ between groups, both reporting a mean of 2.70 with no statistical significance. Estimated glomerular filtration rate was higher in the residual hyperlipidemia group, 75.55 (22.95) mL/min/1.73 m², compared with 69.01 (24.93) mL/min/1.73 m², but this difference did not reach statistical significance. Gender distribution was similar between groups, with males accounting for 902,407 (63%) in the residual hyperlipidemia group and 456,823 (61%) in the non-residual group. Race and ethnicity distributions were comparable across groups, with non-Hispanic White participants representing the largest proportion in both groups. Educational attainment did not differ significantly between groups. Chronic kidney disease was less frequent among participants with residual hyperlipidemia, 373,024 (26%), compared with 302,497 (41%) in those without residual hyperlipidemia, and this difference was statistically significant.

Table 2 presents the adjusted associations between selected factors and residual hyperlipidemia.

**Table 2:** Adjusted ORs for factors associated with residual hyperlipidemia (non-HDL ≥100 mg/dL) Adjusted ORs were derived from survey-weighted logistic regression models. Values are presented as OR with 95% CI and p-values. The asterisk (*) denotes statistical significance (p<0.05). CKD was defined as an eGFR <60 mL/min/1.73 m². The table was generated by the authors using Stata Version 18 (StataCorp LLC, College Station, Texas, United States) [22]. ORs: odds ratios; CI: confidence interval; CKD: chronic kidney disease; eGFR: estimated glomerular filtration rate; GED: general educational development; HDL: high-density lipoprotein

Variable	Adjusted OR (95% CI)	P-value
Age (years)	0.94 (0.91-0.97)	0.001*
Body mass index (kg/m²)	1.06 (1.02-1.10)	0.006*
Income-to-poverty ratio	1.00 (0.82-1.22)	0.989
Gender (female vs. male)	0.83 (0.43-1.60)	0.562
Race/ethnicity		
Mexican American vs. non-Hispanic White	0.70 (0.32-1.54)	0.371
Other Hispanic vs. non-Hispanic White	0.92 (0.43-1.99)	0.834
Non-Hispanic Black vs. non-Hispanic White	0.60 (0.32-1.13)	0.110
Non-Hispanic Asian vs. non-Hispanic White	0.52 (0.23-1.15)	0.106
Other race vs. non-Hispanic White	0.89 (0.27-2.96)	0.852
Education		
Less than high school vs. college or above	1.77 (0.99-3.17)	0.053
High school graduate/GED vs. college or above	1.19 (0.64-2.21)	0.567
CKD: yes vs. no	0.75 (0.40-1.42)	0.374

From the findings above, it is evident that increasing age was associated with lower odds of residual hyperlipidemia (aOR=0.94; 95% CI: 0.91-0.97; p=0.001). Higher body mass index was associated with higher odds of residual hyperlipidemia, with an adjusted odds ratio of 1.06 and a statistically significant p-value (p=0.006). The income-to-poverty ratio was not associated with residual hyperlipidemia. Gender showed no significant association, with females having similar odds compared with males. Race and ethnicity categories did not demonstrate statistically significant differences when compared with non-Hispanic White participants. Educational attainment showed no statistically significant association, although participants with less than a high school education had higher odds that approached statistical significance. Chronic kidney disease was not significantly associated with residual hyperlipidemia.

**Figure 1:**
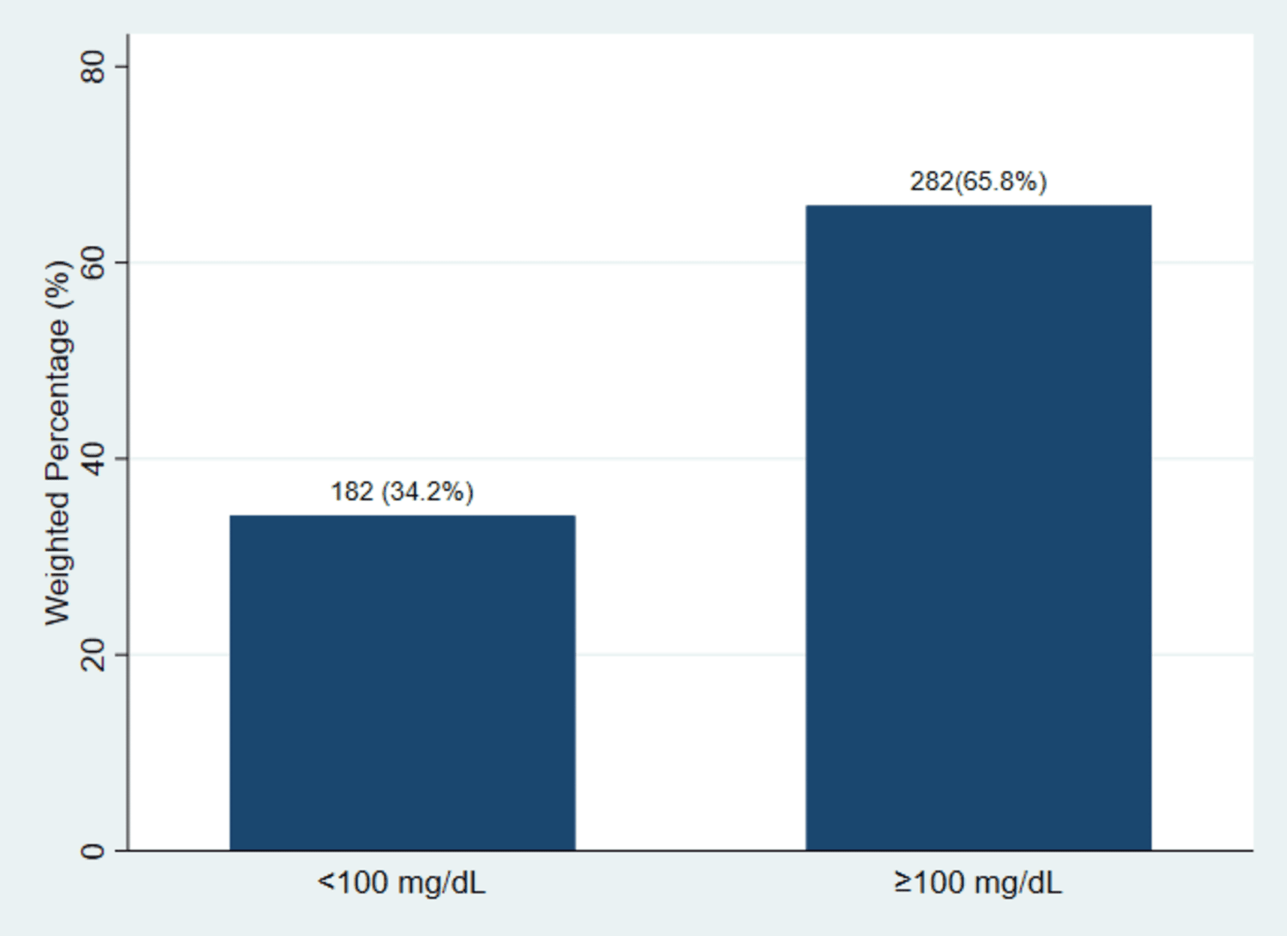
Prevalence of residual hyperlipidemia based on non-high-density lipoprotein cholesterol levels among statin-treated adults with diabetes and atherosclerotic cardiovascular disease (n=464) Counts (N) represent unweighted sample sizes; percentages are survey-weighted estimates.

Figure 1 illustrates the prevalence of residual hyperlipidemia in the study population.

The results indicate that 65.8% of participants had residual hyperlipidemia, while 34.2% had non-HDL-C levels below 100 mg per deciliter. This shows that more than half of statin-treated adults with diabetes and ASCVD had lipid levels above the recommended threshold.

## Discussion

In this study, residual hyperlipidemia was common among statin-treated adults with diabetes and ASCVD. More than half of the population had non-HDL-C above the recommended threshold. Increasing age was associated with lower odds of residual hyperlipidemia, while higher body mass index was associated with increased odds. Other factors, including sex, race and ethnicity, income-to-poverty ratio, education, and chronic kidney disease, were not significantly associated with the outcome. These findings indicate that despite statin therapy, a considerable proportion of high-risk individuals continue to have elevated atherogenic lipoprotein levels.

These findings are consistent with prior evidence showing that residual dyslipidemia persists even among individuals receiving statin therapy [6,7]. Statins remain the cornerstone of lipid-lowering treatment in ASCVD, yet they do not fully address all lipid abnormalities, particularly those related to triglyceride-rich lipoproteins and non-HDL-C [4,8]. Previous studies have reported that individuals with diabetes are more likely to exhibit persistent lipid abnormalities despite treatment, which contributes to ongoing cardiovascular risk [14,15]. The higher prevalence of residual hyperlipidemia observed in this study aligns with these reports and reflects the complex lipid profile seen in diabetes. The association between higher body mass index and residual hyperlipidemia may reflect the metabolic effects of adiposity, including increased production of atherogenic lipoproteins and impaired lipid clearance [15]. In contrast, the inverse association with age may be related to differences in treatment patterns, survival effects, or changes in metabolic profiles with aging, although this relationship requires further evaluation. The lack of significant associations for socioeconomic and demographic variables suggests that clinical factors may play a more prominent role in residual lipid abnormalities within this high-risk group.

Current US guidelines emphasize aggressive lipid lowering in individuals with diabetes and established ASCVD. The American College of Cardiology and American Heart Association guideline recommends an LDL-C target of less than 70 mg per deciliter in very-high-risk individuals and supports the use of additional lipid-lowering therapies when this target is not achieved with statins alone [22]. Similarly, diabetes-specific recommendations highlight the importance of comprehensive lipid management beyond LDL-C, including the use of non-HDL-C as a secondary target [5]. These recommendations support the use of non-HDL-C in this study as a clinically meaningful measure of residual risk. The high prevalence of residual hyperlipidemia observed here suggests that many patients may require further intensification of therapy to achieve recommended lipid targets.

Patients with diabetes and ASCVD represent a very-high-risk population requiring aggressive lipid management [5]. Current guidelines recommend high-intensity statin therapy to achieve an LDL-C reduction of ≥50% from baseline and an LDL-C goal of 55 mg/dL (1.4 mmol/L), with a non-HDL-C goal of 85 mg/dL (2.2 mmol/L) [25,26]. When these targets are not met on maximally tolerated statin therapy, treatment intensification with non-statin agents is indicated [5,26].

American and European guidelines recommend adding ezetimibe, followed by PCSK9 inhibitors if needed, for very-high-risk patients with diabetes and ASCVD on maximally tolerated statins who have not achieved a 50% LDL-C reduction or have LDL-C above 70 mg/dL [27]. Ezetimibe lowers LDL-C by 15-20% and, in the IMPROVE-IT trial (NCT00202878), reduced myocardial infarctions by 24% and ischemic stroke by 39% in participants with diabetes [28]. PCSK9 inhibitors (evolocumab and alirocumab) reduce LDL-C by about 60% and have shown reductions in cardiovascular events in large trials involving patients with diabetes [28].

For patients with moderate hypertriglyceridemia despite maximally tolerated statin therapy, consider icosapent ethyl (highly purified eicosapentaenoic acid) at 4 g daily [26-28]. The REDUCE-IT trial (NCT01492361) showed a 30% reduction in cardiovascular events with icosapent ethyl in statin-treated patients with established ASCVD or diabetes plus additional risk factors and moderate triglycerides [28]. Fibrates lower triglycerides by 30-50% but provide limited cardiovascular benefit when added to statins in patients with diabetes [26]. Multiple trials, including ACCORD, did not demonstrate cardiovascular benefit when fenofibrate was added to statin therapy [28]. Fibrates remain first-line therapy for severe hypertriglyceridemia (≥500 mg/dL) to prevent acute pancreatitis. Fenofibrate is preferred over gemfibrozil when combined with statins due to a lower risk of drug interactions [26].

Treatment intensification should be individualized based on baseline lipid levels, cardiovascular risk, patient preferences, and cost [26]. Shared decision-making is essential, especially when considering PCSK9 inhibitors, given their cost, subcutaneous administration, and storage requirements [5]. For patients with both elevated LDL-C and persistent hypertriglyceridemia, a combined approach to address both lipid abnormalities may be appropriate [28].

In populations receiving statin therapy, non-HDL-C is an essential tool in detecting persistent atherogenic lipoprotein anemia and helps guide adjunctive therapeutic options such as ezetimibe, triglyceride-lowering agents, or PCSK9 inhibitors. Non-HDL-C also overcomes the limitations associated with calculated LDL-C, as it remains reliable in the non-fasting state and in the presence of elevated triglycerides. This usefulness in non-fasting conditions increases its clinical value and benefit in real-world practice by demonstrating the true daily atherogenic exposure, staying reliable despite the variability of triglyceride, and allowing patient testing when the opportunity arises without the need for fasting. This helps to enhance patient convenience, enables clinical decisions to be made during the same visit, and ensures improved detection of residual lipid abnormalities that may have been overlooked if LDL-C alone was relied on. These features together emphasize that non-HDL-C is a cheap, practical, and clinically significant tool useful in identifying and managing residual cardiovascular risk in high-risk groups and not merely a harmonizing metric to LDL-C [22,23].

Several biological mechanisms may explain the persistence of residual hyperlipidemia despite statin therapy. Statins primarily reduce LDL-C by inhibiting hepatic cholesterol synthesis, but they have a more modest effect on triglyceride-rich lipoproteins and remnant particles [8]. In individuals with diabetes, insulin resistance promotes increased hepatic very-low-density lipoprotein production and reduced clearance of circulating lipoproteins, contributing to elevated non-HDL-C levels [15]. Adiposity further exacerbates these pathways through increased free fatty acid flux and chronic low-grade inflammation, which can impair lipid metabolism. These mechanisms may explain the observed association between body mass index and residual hyperlipidemia. Persistent lipid abnormalities may therefore reflect underlying metabolic disturbances that are not fully addressed by statin therapy alone.

Strengths and limitations of the study

This study has several strengths and limitations. The use of nationally representative NHANES data enhances generalizability and allows for population-level estimates. The analysis accounted for the complex survey design, including sampling weights, strata, and clusters. However, the cross-sectional design limits the ability to assess temporal relationships. Several variables, including diabetes, ASCVD, and medication use, were based on self-report and may be subject to misclassification. Missing data required the exclusion of some participants, which may introduce bias. Important factors such as statin intensity, medication adherence, duration of therapy, dietary patterns, and additional lipid-lowering treatments were not available and could not be included in the analysis. Future research should incorporate longitudinal designs and more detailed clinical data to better characterize determinants of residual hyperlipidemia and evaluate strategies to reduce persistent cardiovascular risk.

## Conclusions

This study highlights that residual hyperlipidemia remains common among statin-treated adults with diabetes and ASCVD. A substantial proportion of individuals continue to have elevated atherogenic lipoproteins despite treatment. Higher body mass index was associated with a greater likelihood of residual hyperlipidemia, while increasing age was associated with a lower likelihood. Other demographic and clinical factors showed limited association. These findings point to persistent gaps in lipid control in a high-risk population. Attention to weight-related factors and broader lipid profiles may be important in clinical management. Future research should examine longitudinal patterns, treatment intensity, and additional therapies to better address residual lipid abnormalities and reduce cardiovascular risk.
